# Psora

**Published:** 1898-04

**Authors:** 


Psora is that most ancient, most universal, most destructive, and yet most misapprehended chronic miasmatic disease which for many thousands of years has disfigured and tortured mankind.—(Hahnemann in *Chronic Diseases.*)

